# Supplementary material for: Design, Synthesis, Molecular Modeling, and Anticancer Evaluation of New VEGFR-2 Inhibitors Based on the Indolin-2-One Scaffold
Source: Pharmaceuticals (Basel). 2022 Nov 15;15(11):1416. doi: 10.3390/ph15111416 (PMC9698773; doi:10.3390/ph15111416)
Supplement: Supplementary file 1 [file pharmaceuticals-15-01416-s001.zip › pharmaceuticals-2007509-supplementary.pdf]

pharmaceuticals

MDPI

Article

Design, Synthesis, Molecular Modeling, and Anticancer Evaluation of New VEGFR-2 Inhibitors Based on the Indolin-2-One Scaffold

Mohamed A. Abdelgawad [1,*], Alaa M. Hayallah [2,3,*], Syed Nasir Abbas Bukhari [1], Arafa Musa [4], Mohammed Elmowafy [5], Hamdy M. Abdel-Rahman [6,7] and Mohammed K. Abd El-Gaber [6,*]

1 Department of Pharmaceutical Chemistry, College of Pharmacy, Jouf University, Aljouf 72341, Saudi Arabia
2 Pharmaceutical Organic Chemistry Department, Faculty of Pharmacy, Assiut University, Assiut 71526, Egypt
3 Pharmaceutical Chemistry Department, Faculty of Pharmacy, Sphinx University, New Assiut 71515, Egypt
4 Department of Pharmacognosy, College of Pharmacy, Jouf University, Aljouf 72341, Saudi Arabia
5 Department of Pharmaceutics, College of Pharmacy, Jouf University, Aljouf 72341, Saudi Arabia
6 Medicinal Chemistry Department, Faculty of Pharmacy, Assiut University, Assiut 71526, Egypt;
7 Pharmaceutical Chemistry Department, Faculty of Pharmacy, Badr University, Assiut 2014101, Egypt
* Correspondence: mhmdgwd@ju.edu.sa (M.A.A.); alaa_hayalah@yahoo.com (A.M.H.); moh_khalifa_84@yahoo.com (M.K.A.E.-G.)

Citation: Abdelgawad, M.A.; Hayallah A.M.; Bukhari S.N.A.; Musa A.; Elmowafy M.; Abdel-Rahman, H.M.; Abd El-Gaber, M.K. Design, Synthesis, Molecular Modeling, and Anticancer Evaluation of New VEGFR-2 Inhibitors Based on the Indolin-2-One Scaffold. *Pharmaceuticals* **2022**, *15*, x. https://doi.org/10.3390/xxxxx

Academic Editor(s): Mary J. Meegan

Received: 18 October 2022
Accepted: 9 November 2022
Published: 14 November 2022

Publisher's Note: MDPI stays neutral with regard to jurisdictional claims in published maps and institutional affiliations.

Copyright: © 2022 by the authors. Submitted for possible open access publication under the terms and conditions of the Creative Commons Attribution (CC BY) license (https://creativecommons.org/licenses/by/4.0/).

4.2. Biological investigation

4.2.1. In vitro cytotoxic activity assay

Cell Line cells were obtained from the American Type Culture Collection. The cells were cultured using DMEM (Invitrogen/Life Technologies) supplemented with 10% FBS (Hyclone,), 10 ug/ml of insulin (Sigma), and 1% penicillin-streptomycin. All of the other chemicals and reagents were from Sigma or Invitrogen. Plate cells (cells density 1.2 – 1.8 × 10,000 cells/well) in a volume of 100µl complete growth medium + 100 µl of the tested compound per well in a 96-well plate for 24 hours before the MTT assay. The tested compounds were dissolved in DMSO just before the test.

4.2.1.1. Cell culture protocol

The culture medium was removed to a centrifuge tube. The cell layer was briefly rinsed with 5 mL of 0.25% (w/v) Trypsin and 3 mL of 0.53 mM EDTA solution to remove all traces of serum which contains Trypsin inhibitor. Next, 2.0 to 3.0 ml of Trypsin EDTA solution was added to the flask and cells were observed under an inverted microscope until the cell layer was dispersed (usually within 5 to 15 minutes). Then, 6.0 to 8.0 mL of complete growth medium was added and cells aspirated by gently pipetting. The cell suspension was transferred to the centrifuge tube with the medium and cells from step 1, and centrifuged at approximately 125 xg for 5 to 10 minutes. The supernatant was discarded and the cell pellet was suspended in fresh growth medium. Appropriate aliquots of the cell suspension were added to new culture vessels and incubated at 37°C for 24 hrs. After treatment of cells with the serial concentrations of the compound to be tested, incubation was carried out for 48 h at 37ºC. The plates were examined under an inverted microscope and proceeded for the MTT assay

4.2.1.2. MTT-Cytotoxicity assay protocol

MTT assay protocol was applied as previously described to assess the anti-proliferative activity of the synthesized compounds. Two human cancer cell lines (MCF-7 and HepG-2) were used in this test. At first, the cell lines were cultured in RPMI-1640 medium

with 10% fetal bovine serum. Antibiotics (100 units/ml penicillin and100 µg/ml streptomycin) were added at 37°C in a 5% CO_2 incubator. The cell lines were seeded in a 96-well plate at a density of 1.0 x 104 cells / well at 37 °C for 48 h under 5% CO_2. After incubation, the cells were treated with different concentrations of the synthesized compounds and incubated for 24 h. After 24 h of drug treatment, 20 µl of MTT solution (5mg/ml) was added and incubated for 4 h. 100 µl of dimethyl sulfoxide (DMSO) was added into each well to dissolve the formed purple formazan. The colored solution was measured and recorded at an absorbance of 570 nm using a plate reader (EXL 800, USA). The relative cell viability was calculated in percentage as (A570 of treated samples/A570 of untreated sample) X 100. The half maximal inhibitory concentration (IC_{50}) values were presented using non-linear regression analysis of the data set from three experiments, with three plate wells used for an individual concentration.

4.2.2. In vitro VEGFR-2 kinase inhibitory assay

The most potent anti-proliferative derivatives, **5b, 10e, 10g, 15a,** and **17a,** were tested for their inhibitory activity against VEGFR-2. Human BPS bioscience® VEGFR2 (KDR) Kinase Assay Kit (Enzyme-Linked Immunosorbent Assay) was utilized in this test. The luminescent signal from the assay is correlated with the amount of ATP present and is inversely correlated with the amount of kinase activity. The compounds were diluted to 100 µM in 10% DMSO and 5 ml of the diluted solution was added to a 50 ml reaction so that the final concentration of DMSO was 1% in all of reactions. All of the enzymatic reactions were conducted at 30 °C for 40 min. The 50 µl reaction mixture contains 40 µM Tris, pH 7.4, 10 µM $MgCl_2$, 0.1 mg/ml BSA, 0.2 mg/ml Poly (Glu, Tyr) substrate, 10 µM ATP, and VEGFR-2. After the enzymatic reaction, 50 ml of Kinase-GloPlus Luminescence kinase assay solution (Promega) was added to each reaction and the plate was incubated for 5 min at room temperature. Luminescence signal was measured using a BioTek Synergy 2 microplate reader. The protein kinase assays used to determine IC_{50} values were performed using a ADP-GloTM assay kit from Promega, which measures the generation of ADP by the protein kinase. Generation of ADP by the protein kinase reaction leads to an increase in luminescence signal in the presence of the ADP-GloTM assay kit. The assay was started by incubating the reaction mixture in a 96-well plate at 30 °C for 30 min. After the 30 min incubation period, the assay was terminated by the addition of 25 ml of ADP-GloTM Reagent (Promega). The 96-well plate was shaken and then incubated for 40 min at ambient temperature; 50 ml of Kinase detection reagent was added, the 96-well reaction plate was then read using the ADP-Glo Luminescences Protocol on a GloMax plate reader (Promega: Catalog #E7031). The blank control was set up which included all the assay components, except the addition of appropriate substrates (replaced with equal volume of kinase assay buffer). The corrected activity for each protein kinase target was determined by removing the blank control value. IC_{50} determination for inhibitor against VEGFR-2 was estimated by generating a graph of log inhibitor vs normalized response with variables. Simple linear regression analysis was established using VEGFR-2 inhibitory activities as a dependent variable, and anti-proliferative activities as an independent variable of the tested compounds. Such statistical analysis was carried out to check the extent of correlation between anti-proliferative activities and VEGFR-2 inhibitory activities. The results revealed that the coefficients of determination (r^2) were 0.84 and 0.87 against MCF-7 and HepG2 cell lines, respectively.

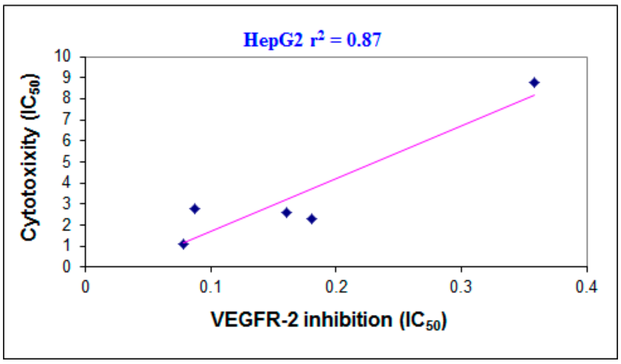

Figure S1. Simple linear regression for the correlation between cytotoxicity against HepG2 cell line and VEGFR-2 inhibition.

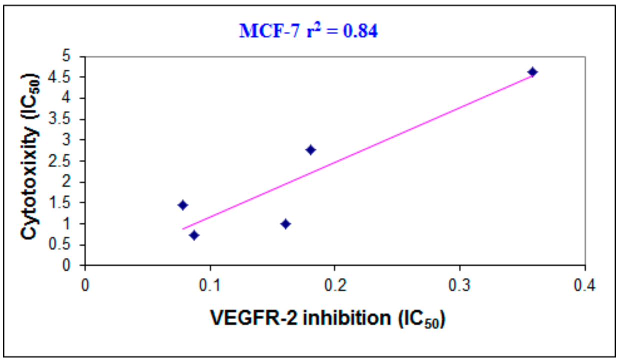

Figure S2. Simple linear regression for the correlation between cytotoxicity against MCF-7 cell line and VEGFR-2 inhibition.

4.2.3. Cell cycle analysis

To determine the role of the synthesized compounds in cell cycle distribution, cell cycle analysis was performed using propidium iodide (PI) staining and flow cytometry analysis for compound **17a**. A Flow Cytometry Kit for Cell Cycle Analysis (ab139418_Propidium Iodide Flow Cytometry Kit/BD) was used in this test. HepG2 cells were treated with compound **17a** (1.13 µM) for 24 h. Then, the cells were fixed in 70% ethanol at 4 °C for 12 h. After that, the cells were washed with cold PBS, incubated with 100 µl RNase A at 37 °C for 30 min, and stained with 400 µl PI in the dark at room temperature for a further 30 min. The stained cells were measured using a BD FACSCalibur™ Flow Cytometer, and the data were analyzed using Flowing software (version 2.5.1, Turku Centre for Biotechnology, Turku, Finland).

4.2.4. Apoptosis analysis

Flow cytometry cell apoptosis analysis was used to investigate the apoptotic effect of the synthesized compounds. HepG2 cells were treated with compound **17a** (1.13 µM) for 24 h, collected by trypsin, centrifuged, washed two successive times with PBS, suspended in 500 µL binding buffer, and double stained with 5 µL Annexin V-FITC and 5 µL PI in the dark at room temperature for 15 min. The stained cells were measured using a BD FACSCalibur™ Flow Cytometer and analyzed using Flowing software.

4.2.5. Caspase-3 and -9 expression assay

The effect of compound **17a** (1.13 µM) for 24 h on caspase-3 and caspase-9 mRNA expression in the HepG2 cell line was determined by real-time PCR analysis using Qiagen RNA extraction/BioRad syber green PCR MMX. Total RNA was extracted from HepG2

cells using an RNeasy Pure mRNA Bead Kit. mRNA was reverse-transcribed, and quantitative PCR was performed using a Rotorgene RT- PCR system. The primers and probes were as follows:

Casp3 F: 5'- GGAAGCGAATCAATGGACTCTGG -3'
Casp3 R: 5'- GCATCGACATCTGTACCAGACC -3'
Casp9 F 5'- GTTTGAGGACCTTCGACCAGCT-3'
Casp9 R 5'- CAACGTACCAGGAGCCACTCTT-3'
β-actin F: 5'-ATCGTGGGGCGCCCCAGGCAC-3'
β-actin R: 5'-CTCCTTAATGTCACGCACGATTTC-3'

Reverse transcription and PCR were performed as a one-step reaction. The reverse transcription conditions were 48 °C for 30 min and AmpliTaq Gold activation at 95 °C for 10 min, and the PCR cycling conditions were 40 cycles at 95 °C for 15 s and 60 °C for 1 min. A relative standard curve was generated and quantification of their caspase-3 and caspase-9, and β-actin mRNAs was determined from the curve. Samples were standardized using the β-actin signal and results were expressed as fold changes relative to the non-stimulated sample at 0 h.

4.2.6. Bax and Bcl-2 expression assay

The expression of BAX and Bcl-2 proteins were determined using Western blot analysis. In brief, HepG2 cells were treated with control or with the synthesized compound **17a** (1.13 μM). Then cells were lysed in 250 μL precold lysis buffer (pH 7.4: Tris-Base [10 mM], NaCl [100 mM], ethylenediaminetetraacetic acid [EDTA, 25 mM], ethylene glycol bis (2- aminoethyl) tetraacetic acid [EGTA, 25 mM], 1% [v/v] NP-40, and 1% [v/v] Triton X-100) supplemented with 1:350 protease:phosphatase inhibitors cocktail (Sigma). The cells were immediately frozen at −20°C for 1.5 hours for further analysis, collected by cell scraper, sonicated 3 × 10 seconds, and centrifuged (13000 rpm, 15 minutes). Total protein concentrations in the supernatant were colorimetrically determined using the Pierce 660 nm Protein Assay method (Thermo Fisher Scientific, Rockford, IL), with BSA as the standard. Equal amounts of protein (25 μg) samples were mixed with SDS-loading buffer (pH 6.8: Tris-HCl [700 mM], dithiothreitol [DTT, 600 mM], sodium dodecyl sulfate [SDS, 12%], glycerol [60%], and bromophenol blue [0.012%]), denatured by boiling at 95°C for 10 minutes, allowed to cool on ice for 15 minutes, vigorously vortexed for 30 seconds, loaded into SDS-polyacrylamide gel and separated by an electrophoresis unit (Cleaver Scientific Ltd, UK), and transferred onto polyvinylidene fluoride membranes (Bio-Rad) for 35 minutes using a Trans-Blot SD semi-dry transfer cell (Bio-Rad) at 250 mA and 22 V. Membranes were blocked with 5% (w/v) blotting grade dry milk (Bio-Rad) in Tris-buffered saline/Tween-20 (TBS-T) (pH 7.5: Tris-base [20 mM], NaCl [150 mM], and 0.05% [v/v] Tween- 20) while shaken for 1.5 hour at RT, and then incubated with the corresponding primary antibody against BAX (Biovision, USA) and Bcl-2 (Bioimaging, system, syngene, UK) for 9-10 hours at 4°C in a humidified chamber. The blots were washed with TBS-T three times for 15 minutes and incubated with matched horseradish peroxidase (HRP)-linked secondary antibodies (Dako, Denmark) for another 1 hour at RT, followed by washing 3 × 15 min with TBS-T. After membranes were incubated at RT with 1:1 reagent mixture of chemiluminescence Western Lightning ECL (Perkin Elmer, Waltham, MA) for 1 minute, the bands were visualized in Chemi- Doc imager (Bio-Rad). Means of the detected blot intensities were then quantified, analyzed by the combined Bio-Rad Image Lab software and their corresponding background subtracted, with normalization to the corresponding bands density of β-actin as the sampling loading control. Data were collected from three separate experiments.

4.3. Molecular modeling studies

4.3.1. Molecular docking study

The molecular modeling calculations and docking simulation studies were carried out on a Dell precision T3600 workstation with Intel Xeon® CPU-1650.0 @ 3.20 GHz and Windows 7 operating system using Molecular Operating Environment software (MOE 2020.09, Chemical Computing Group, Canada). The target compounds were built using the builder interface of the MOE program and subjected to energy minimization and partial charges calculation. The produced molecular model was subjected to a systematic conformational search, where all items were set as default with RMS gradients of 0.01 kcal/mol and RMS distances of 0.1 Å. The X-ray crystallographic structure of VEGFR-2 complexed with sunitinib (PDB Id: 4AGD) was obtained from the Protein Data Bank. The target protein was prepared for docking studies, where: (i) extra chains and unnecessary water molecules were removed from the enzyme active site; (ii) hydrogen atoms were added to the structure with their standard geometry; (iii) MOE Alpha Site Finder was used for the active sites search in the enzyme structure, and dummy atoms were created from the obtained alpha spheres. Docking of the conformation database of the target compounds was carried out by the MOE-Dock tool and using Dummy atoms as the docking site, Triangle matcher as the placement methodology, and London dG as the scoring function. The best pose for each ligand was explored using the LigX tool, and the obtained ligand–enzyme complex model was then used in calculating the energy parameters using MMFF94x force field energy calculation and predicting the ligand–enzyme interactions at the active site.

Spectral data:

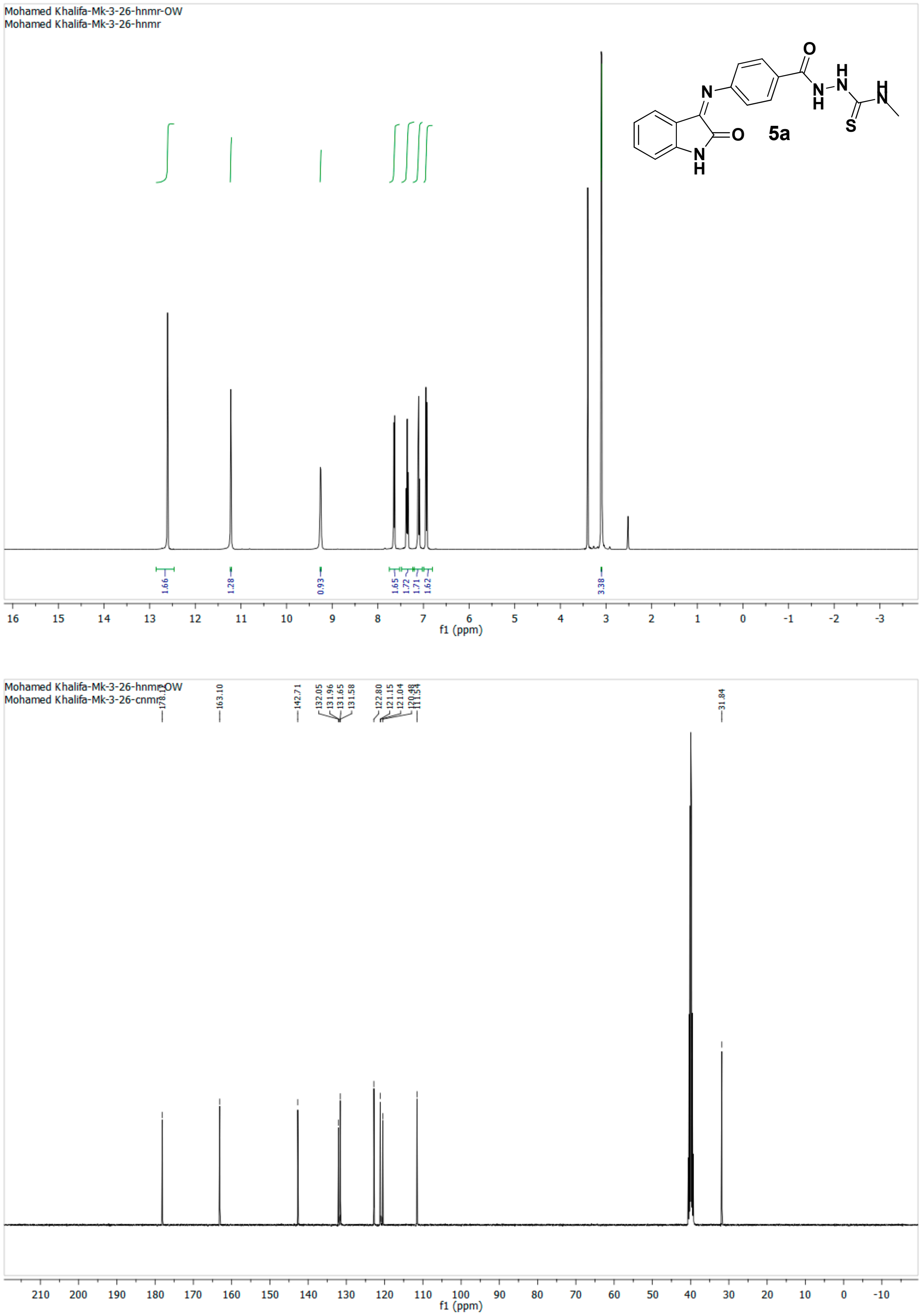

Figure S3. ^{1}H and ^{13}C NMR spectra of compound 5a (DMSO-d6).

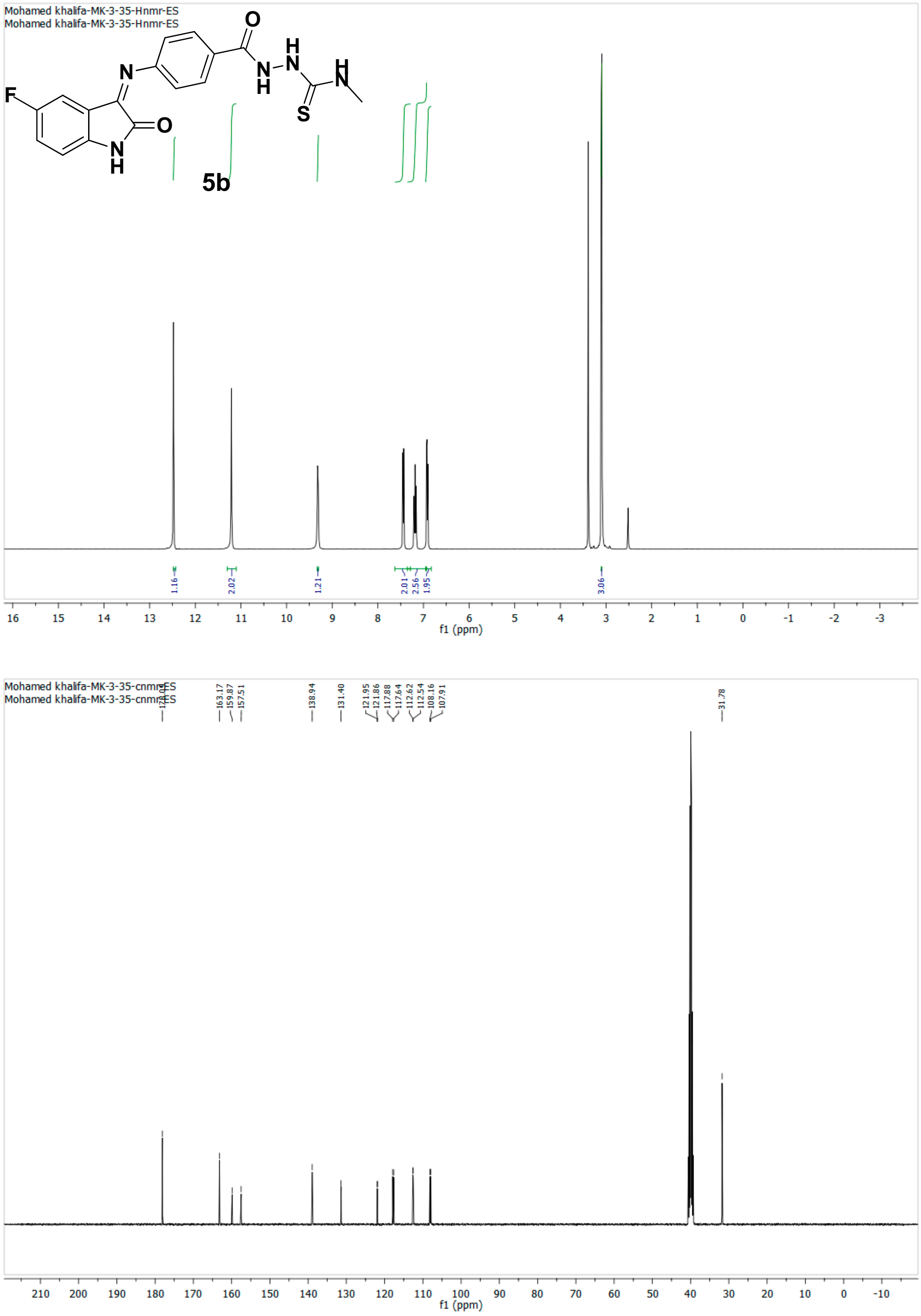

Figure S4. ^{1}H and ^{13}C NMR spectra of compound 5b (DMSO-d6).

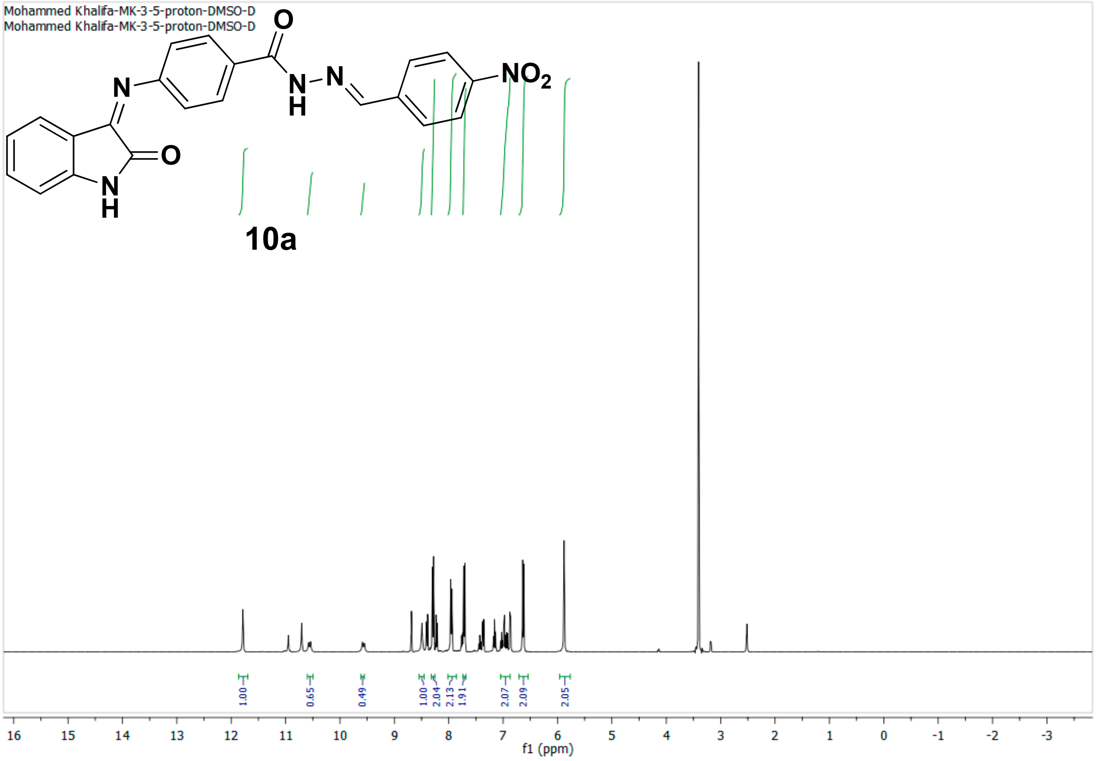

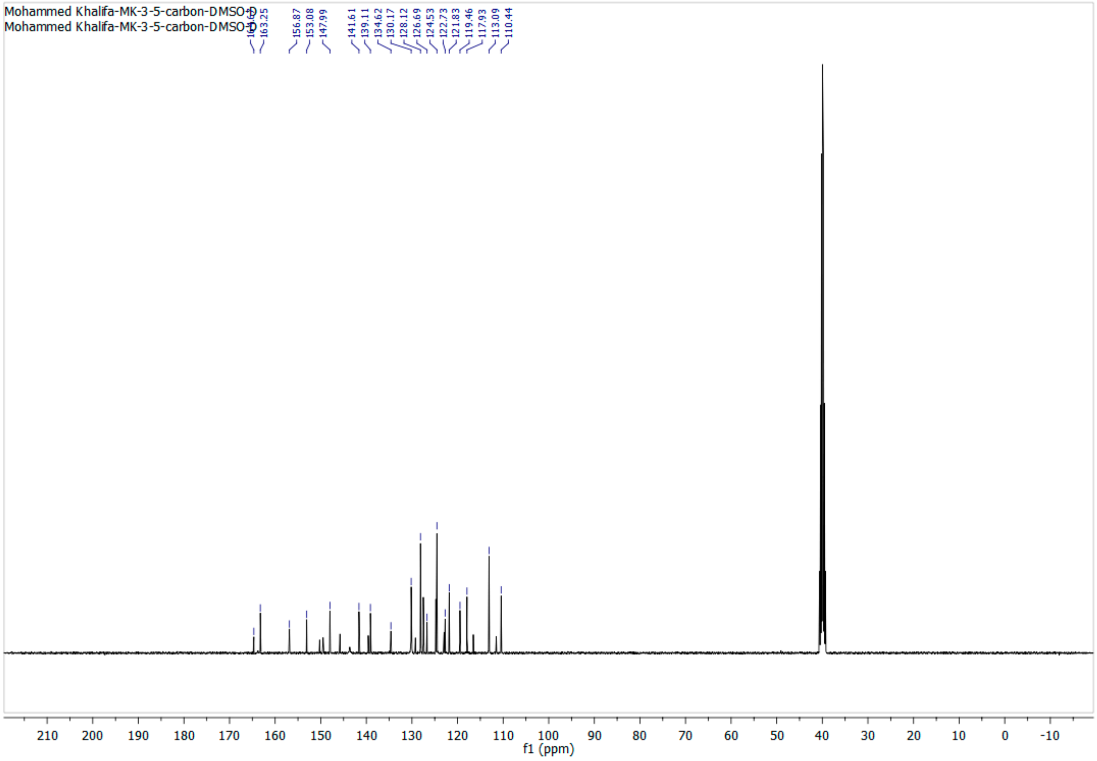

Figure S5. ^{1}H and [13] C NMR spectra of compound 10a (DMSO-d6).

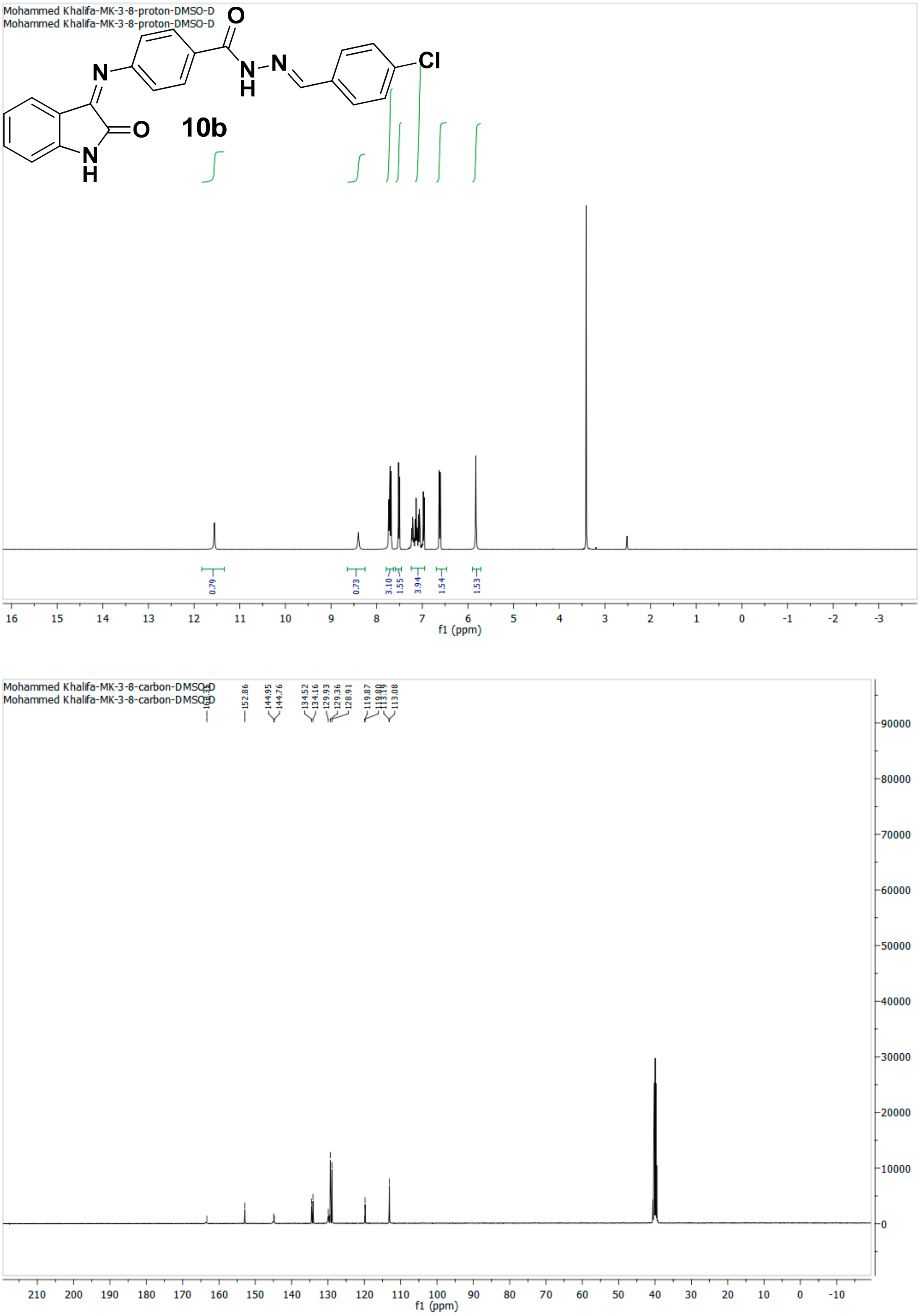

Figure S6. ^{1}H and ^{13}C NMR spectra of compound 10b (DMSO-d6).

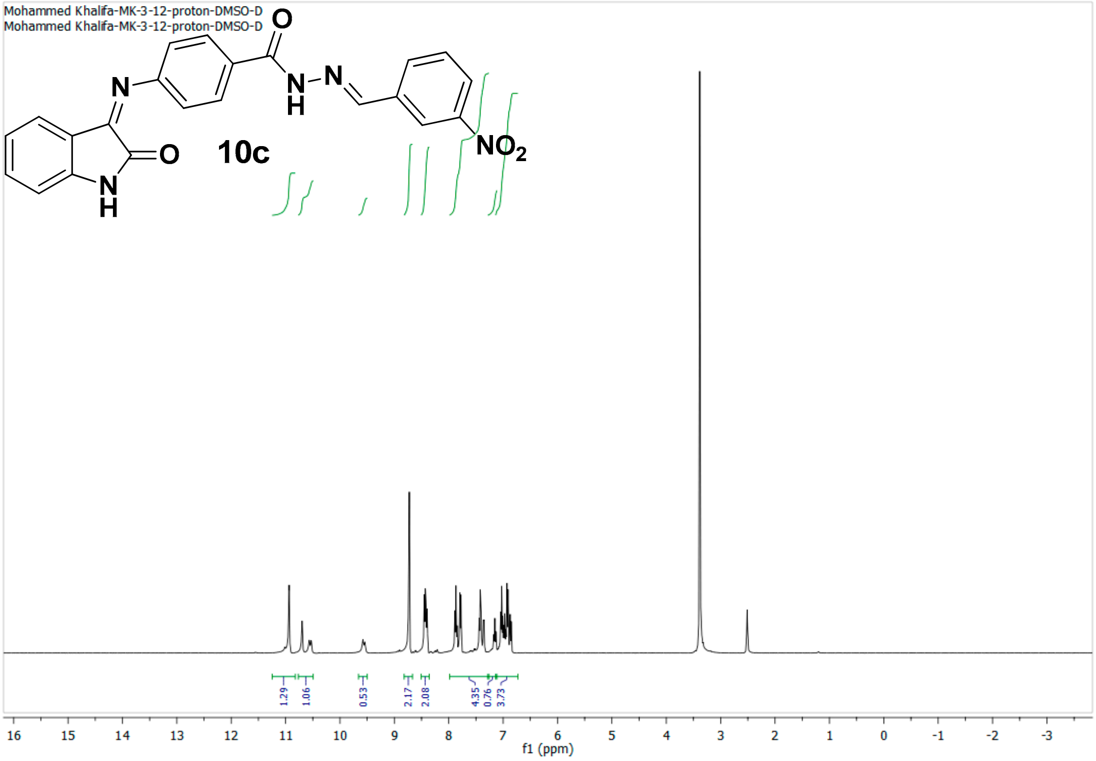

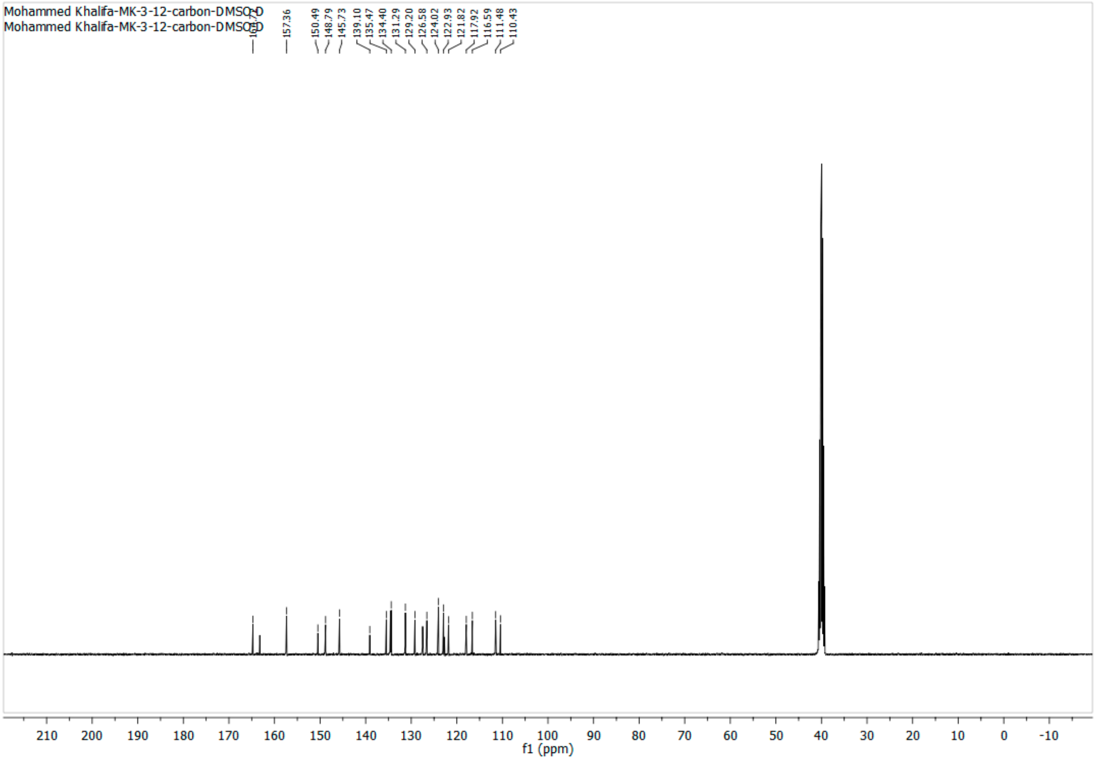

Figure S7. ^{1}H and [13] C NMR spectra of compound 10c (DMSO-d6).

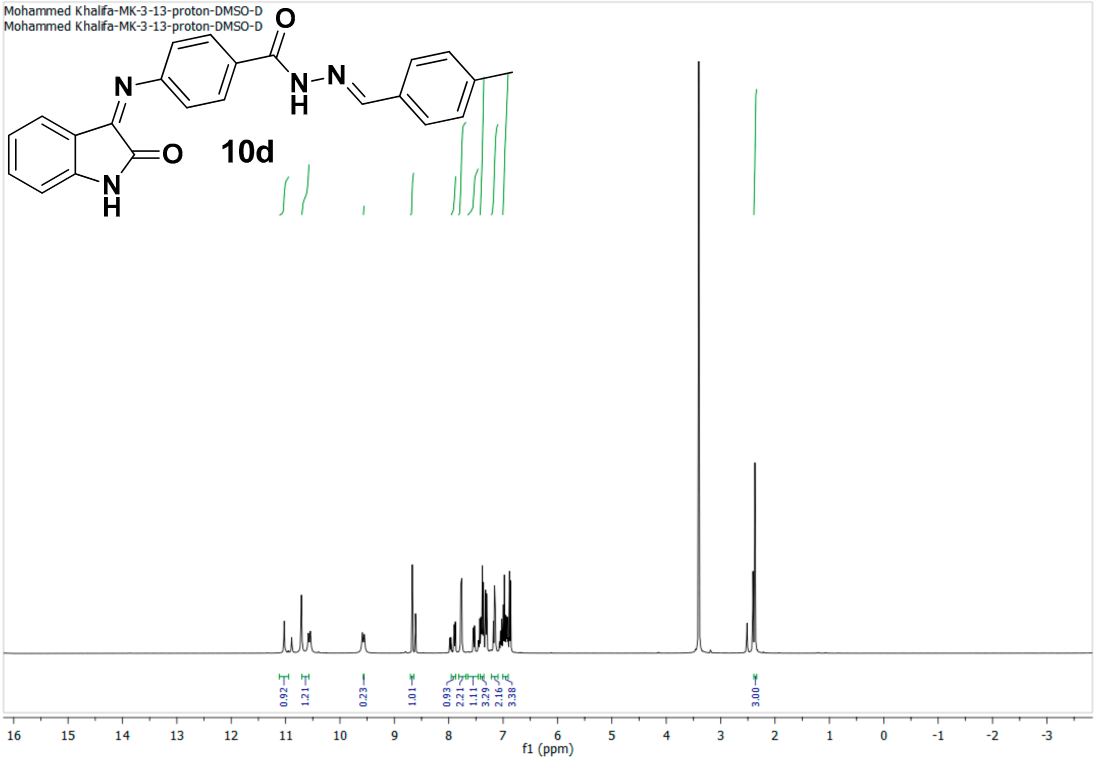

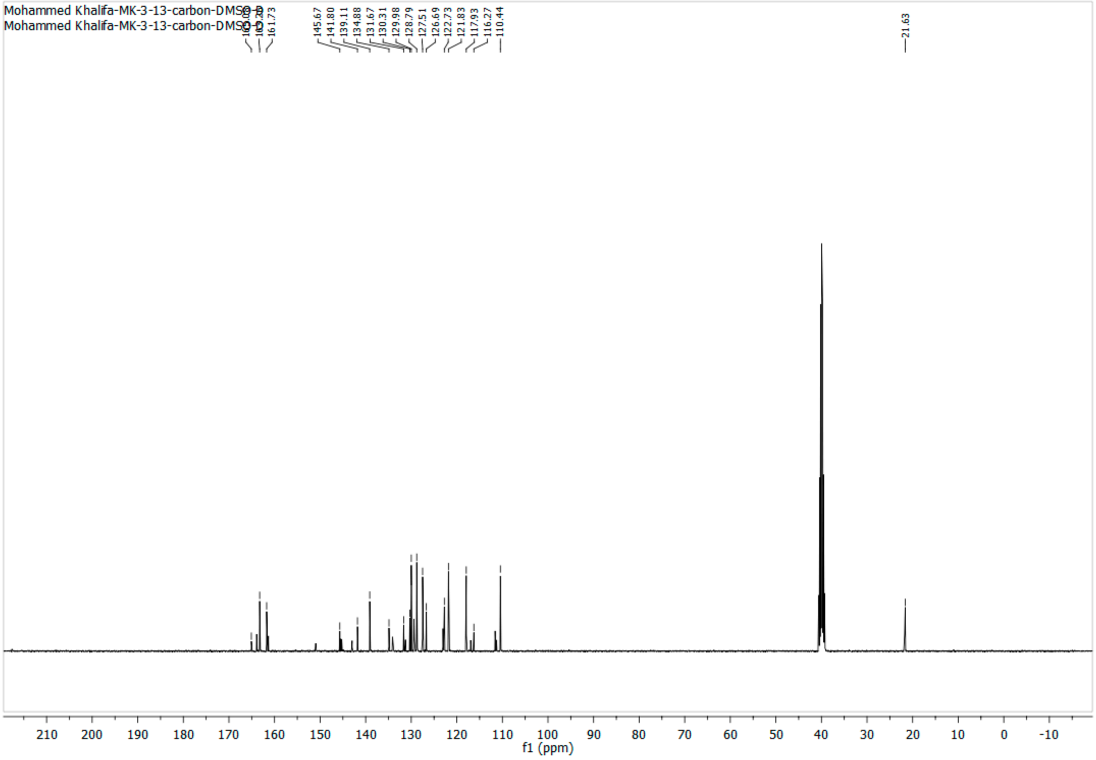

Figure S8. ^{1}H and ^{13}C NMR spectra of compound 10d (DMSO-d6).

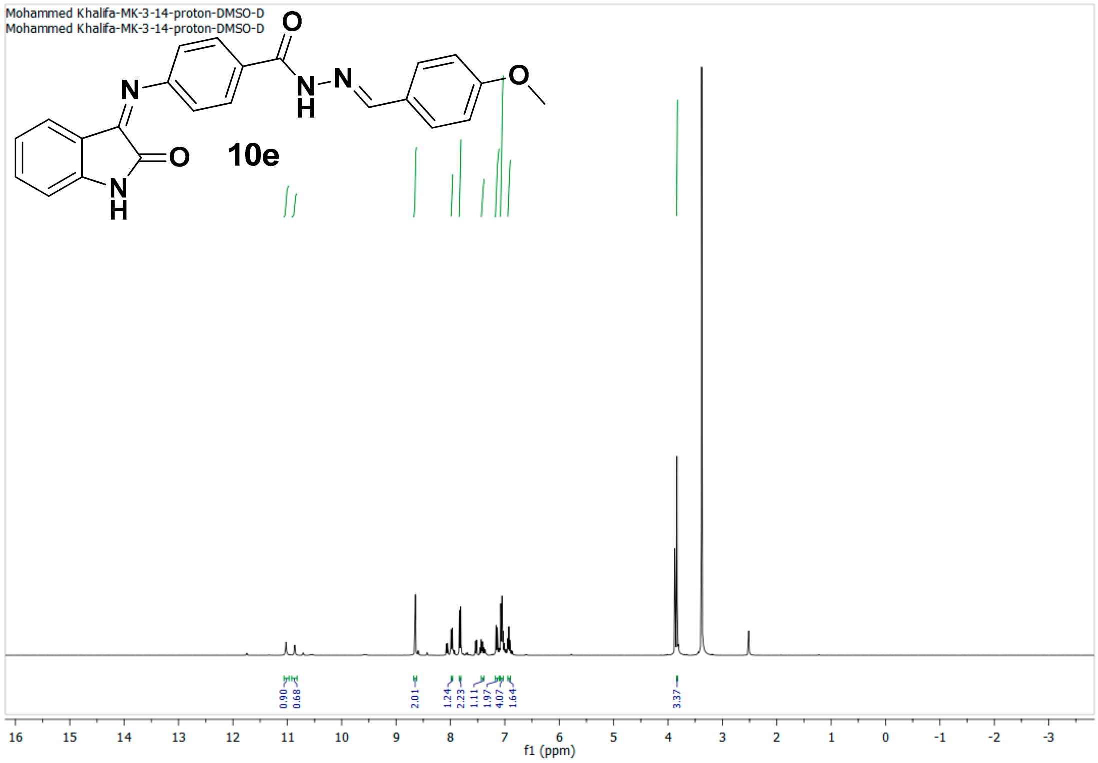

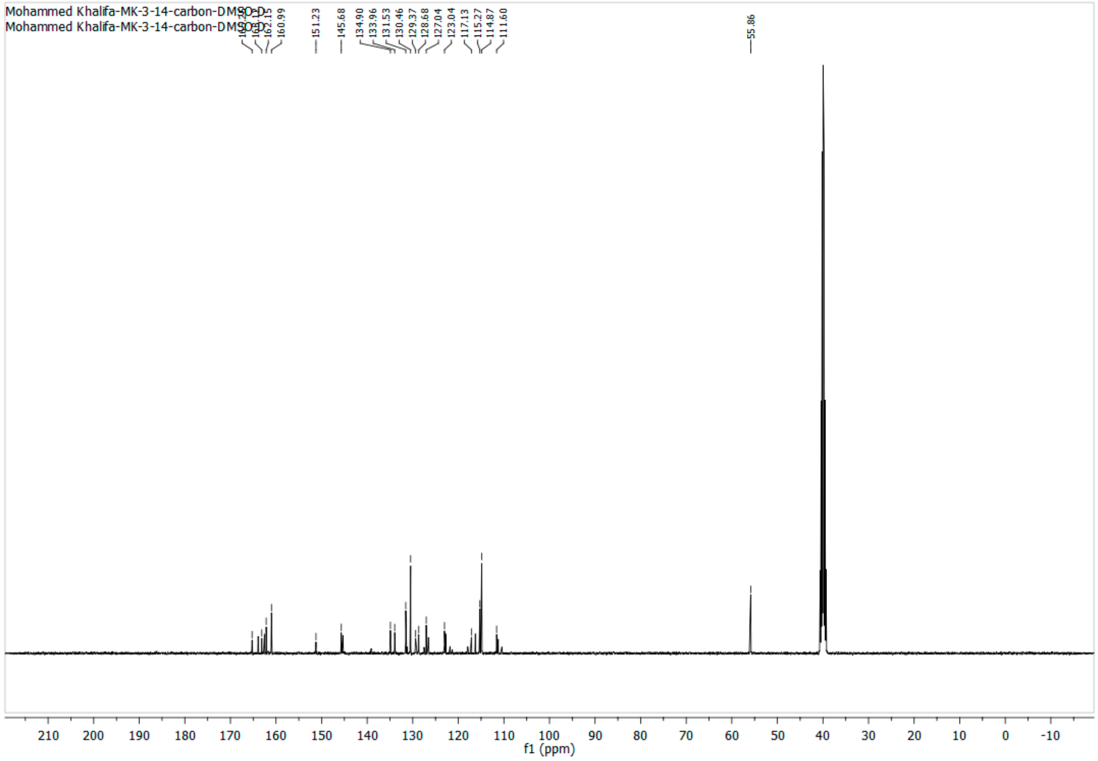

Figure S9. ^{1}H and ^{13}C NMR spectra of compound 10e (DMSO-d6).

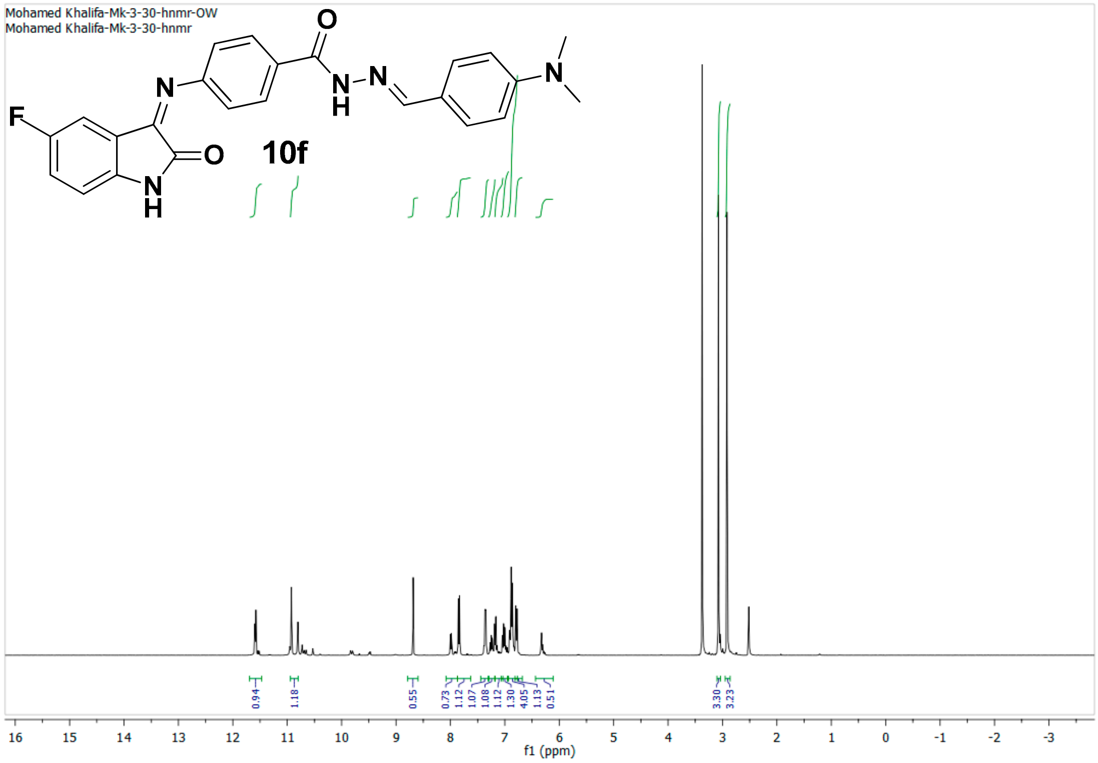

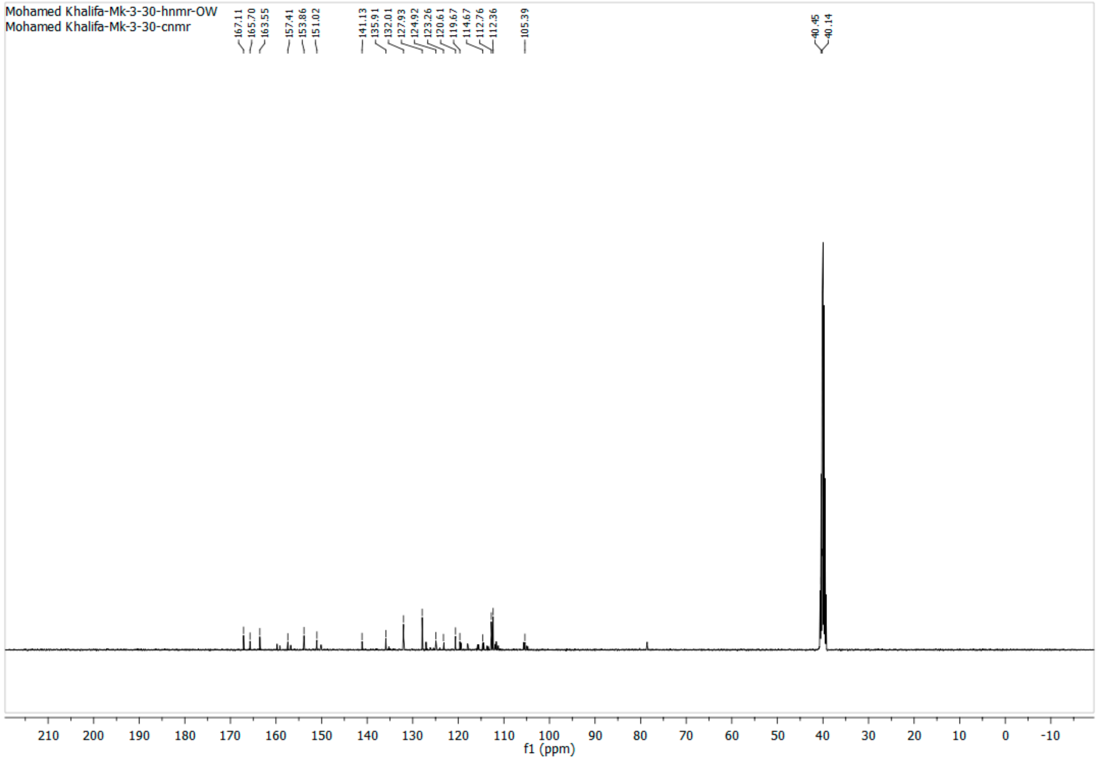

Figure S10. ^{1}H and ^{13}C NMR spectra of compound 10f (DMSO-d6).

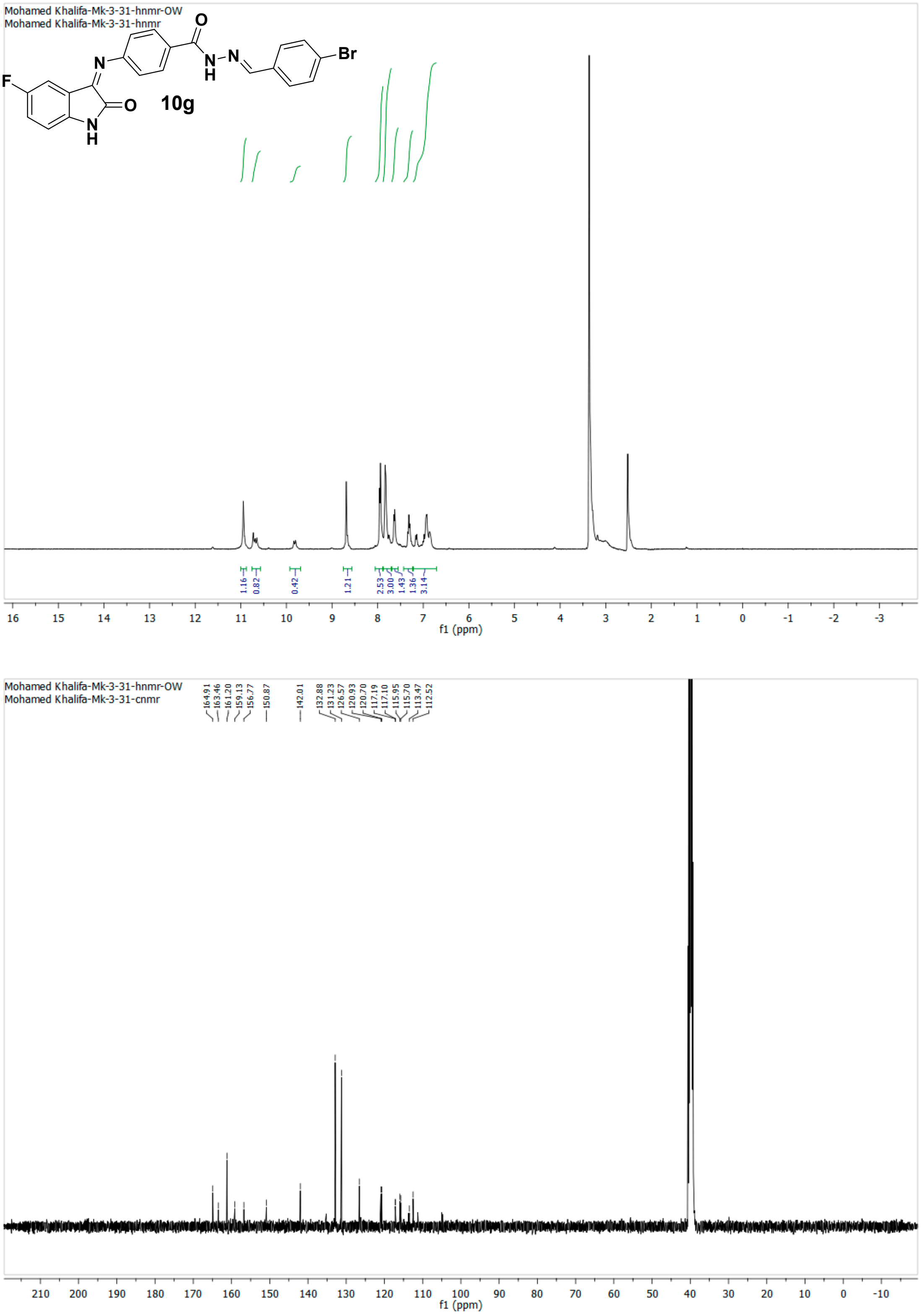

Figure S11. ^{1}H and ^{13} C NMR spectra of compound 10g (DMSO-d6).

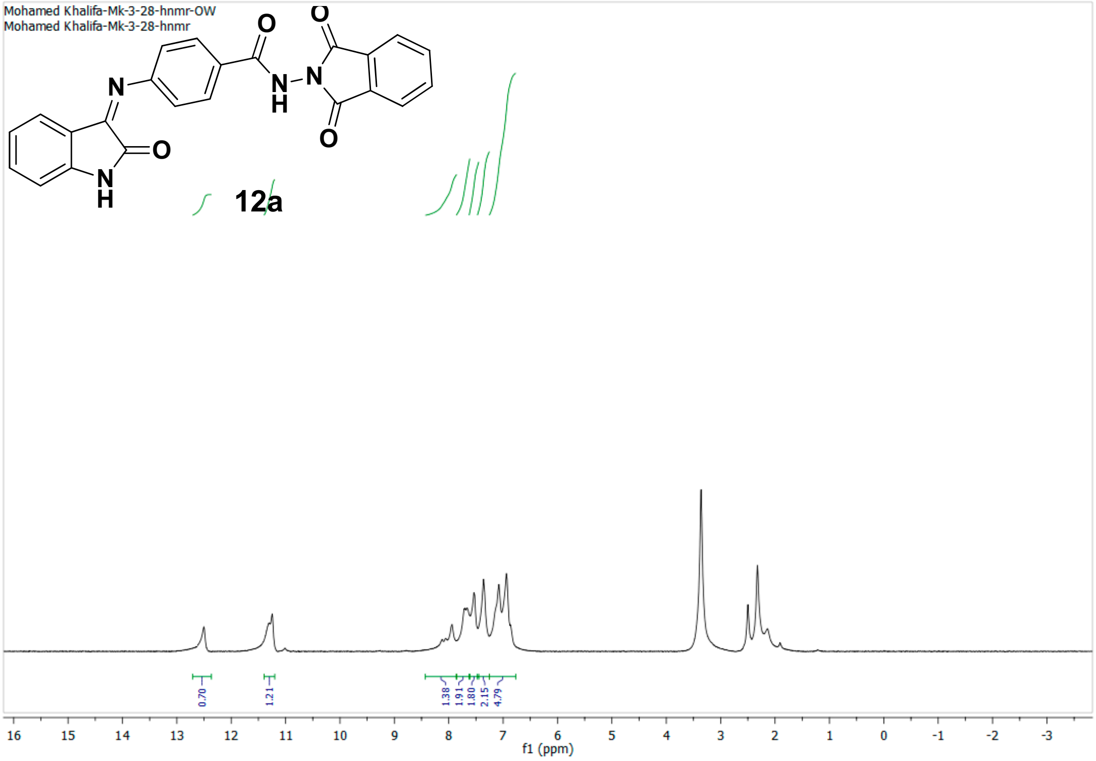

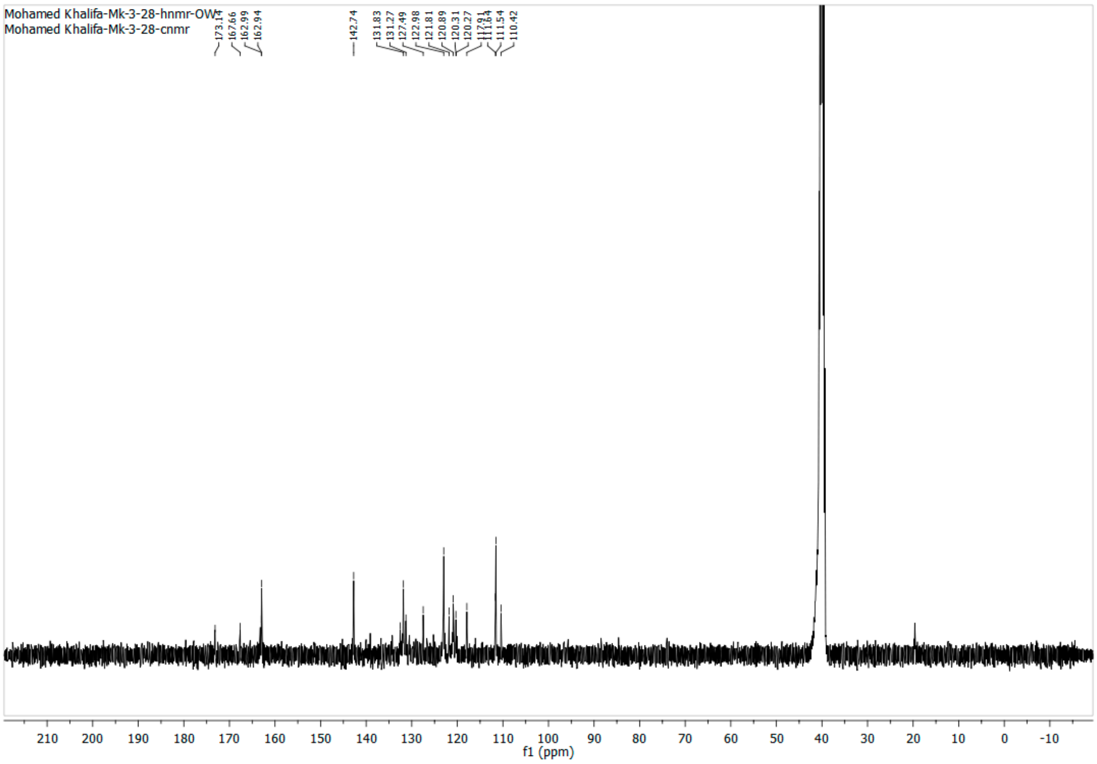

Figure S12. ^{1}H and ^{13}C NMR spectra of compound 12a (DMSO-d6).

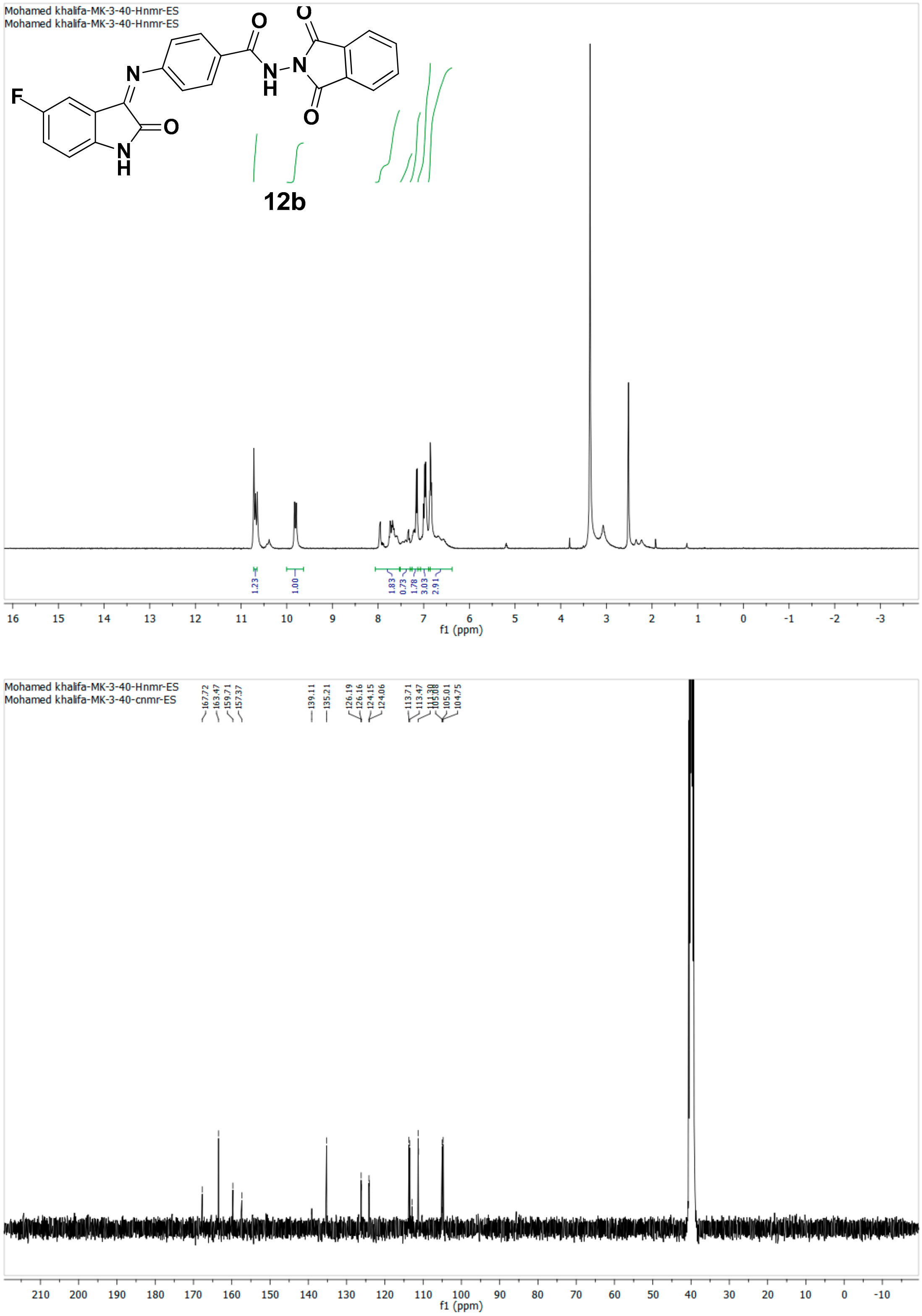

Figure S13. ^{1}H and [13] C NMR spectra of compound 12b (DMSO-d6).

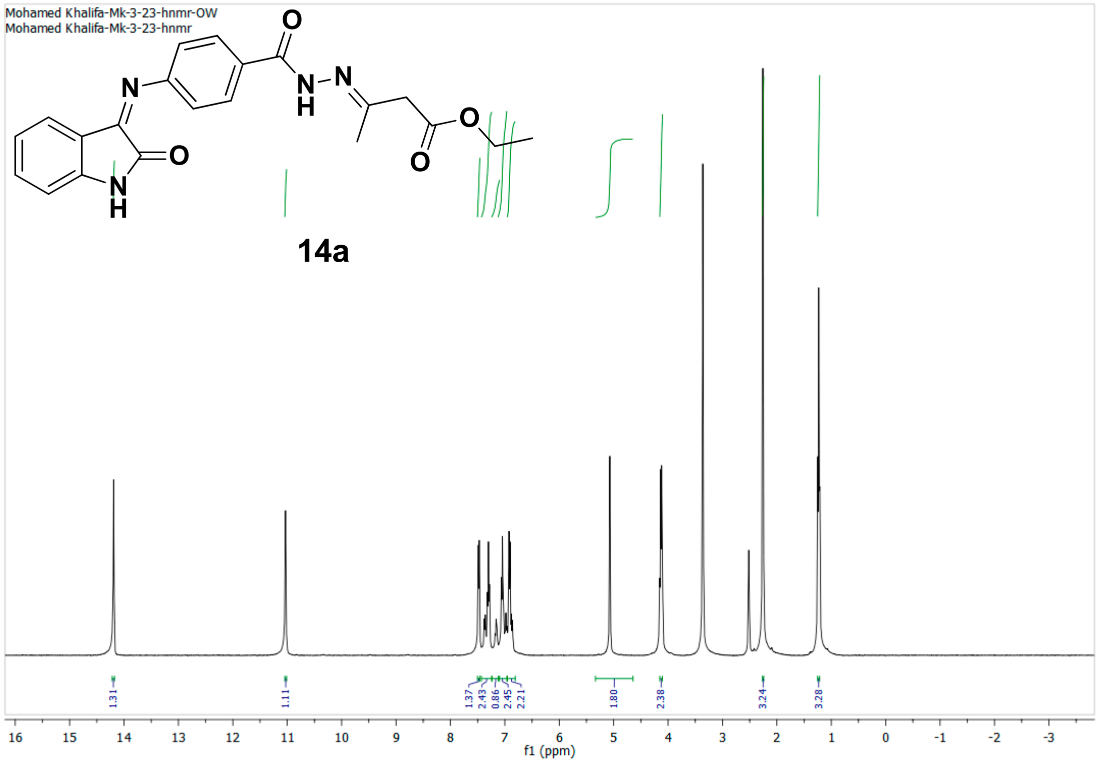

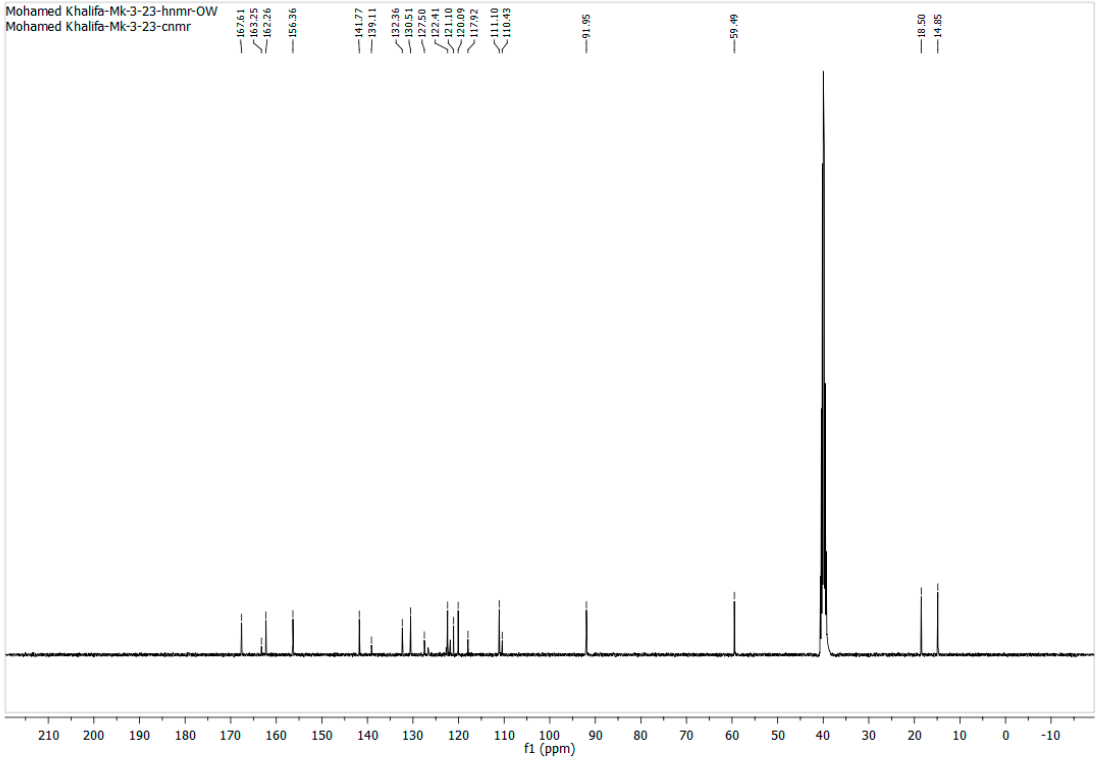

Figure S14. ^{1}H and ^{13}C NMR spectra of compound 14a (DMSO-d6).

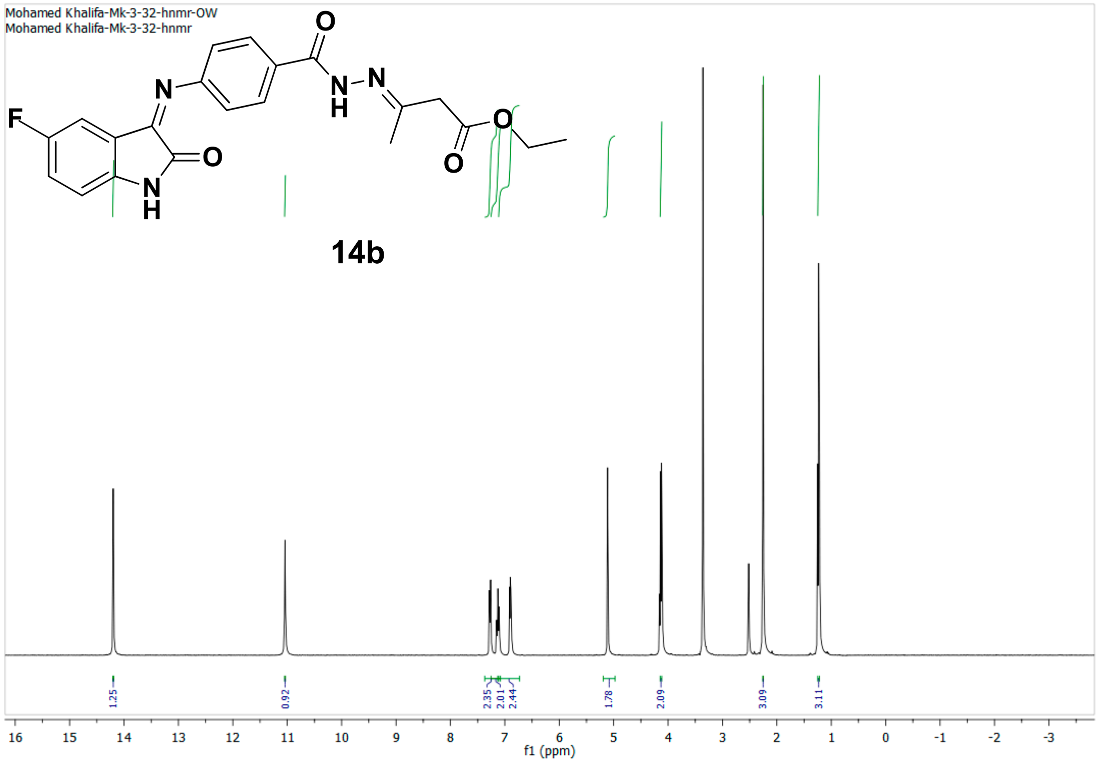

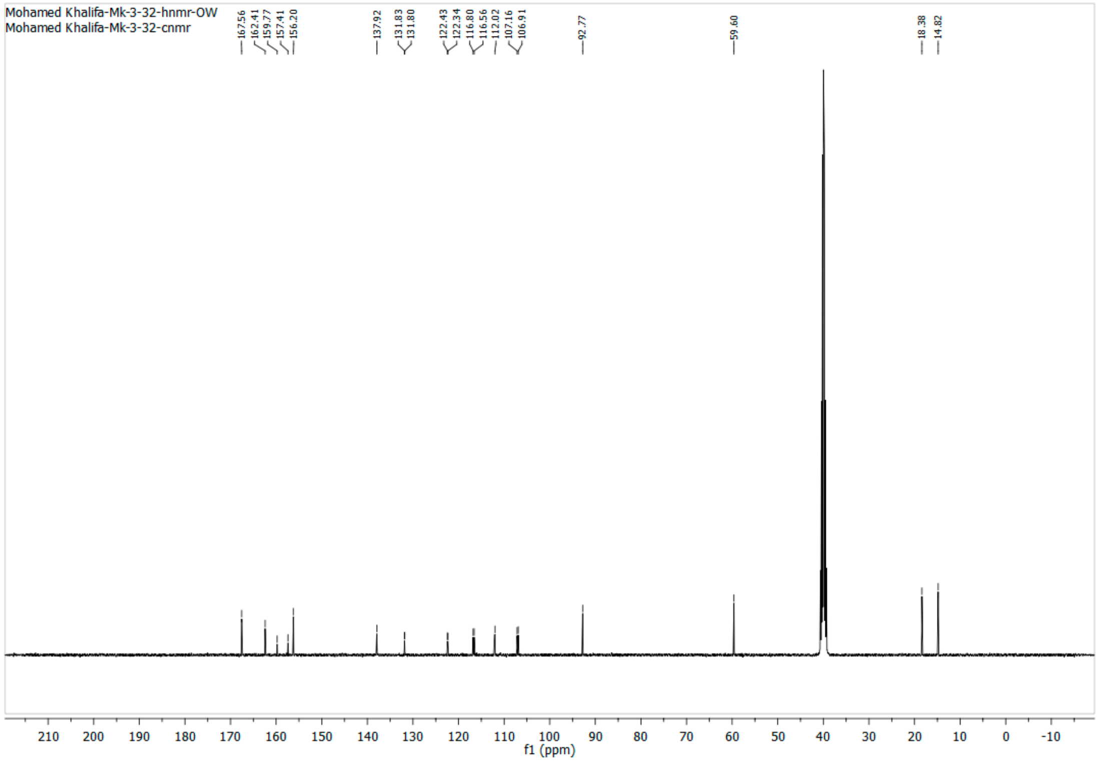

Figure S15. ^{1}H and ^{13}C NMR spectra of compound 14b (DMSO-d6).

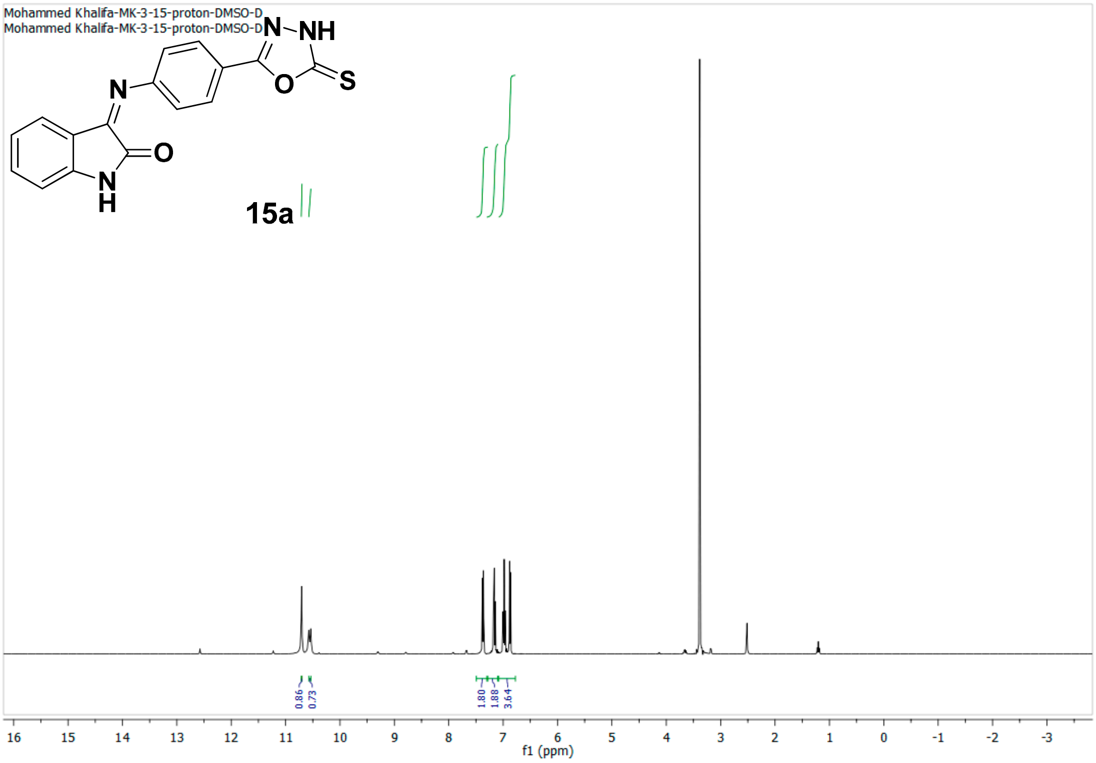

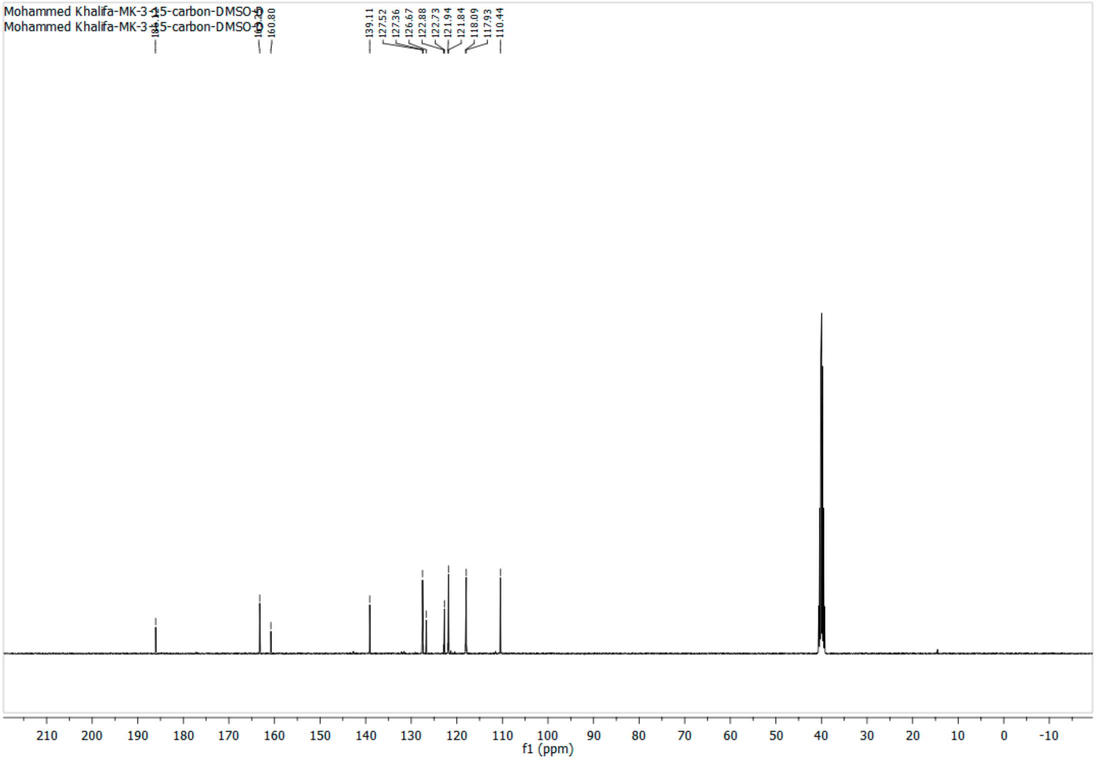

Figure S16. ^{1}H and ^{13}C NMR spectra of compound 15a (DMSO-d6).

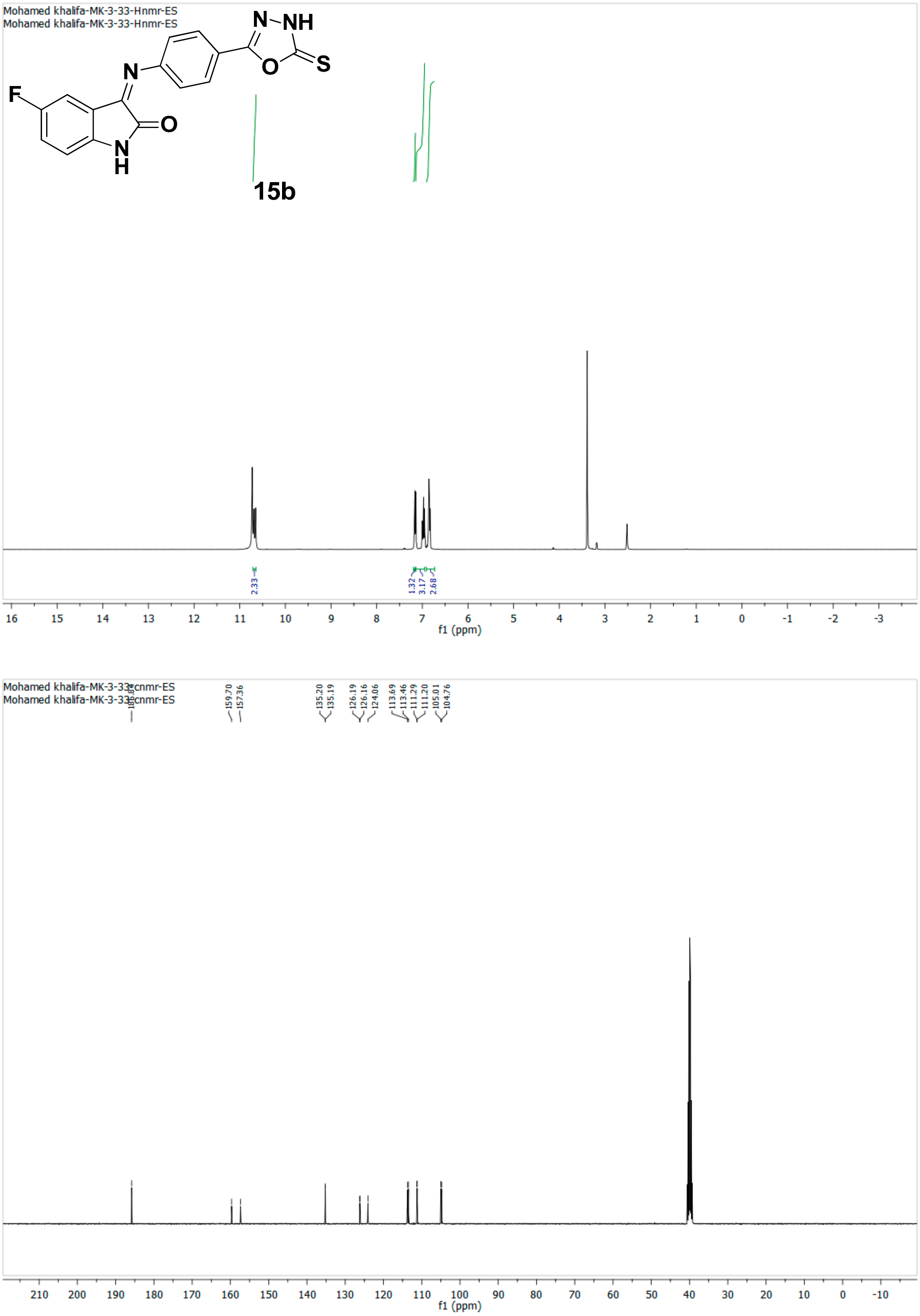

Figure S17. ^{1}H and ^{13}C NMR spectra of compound 15b (DMSO-d6).

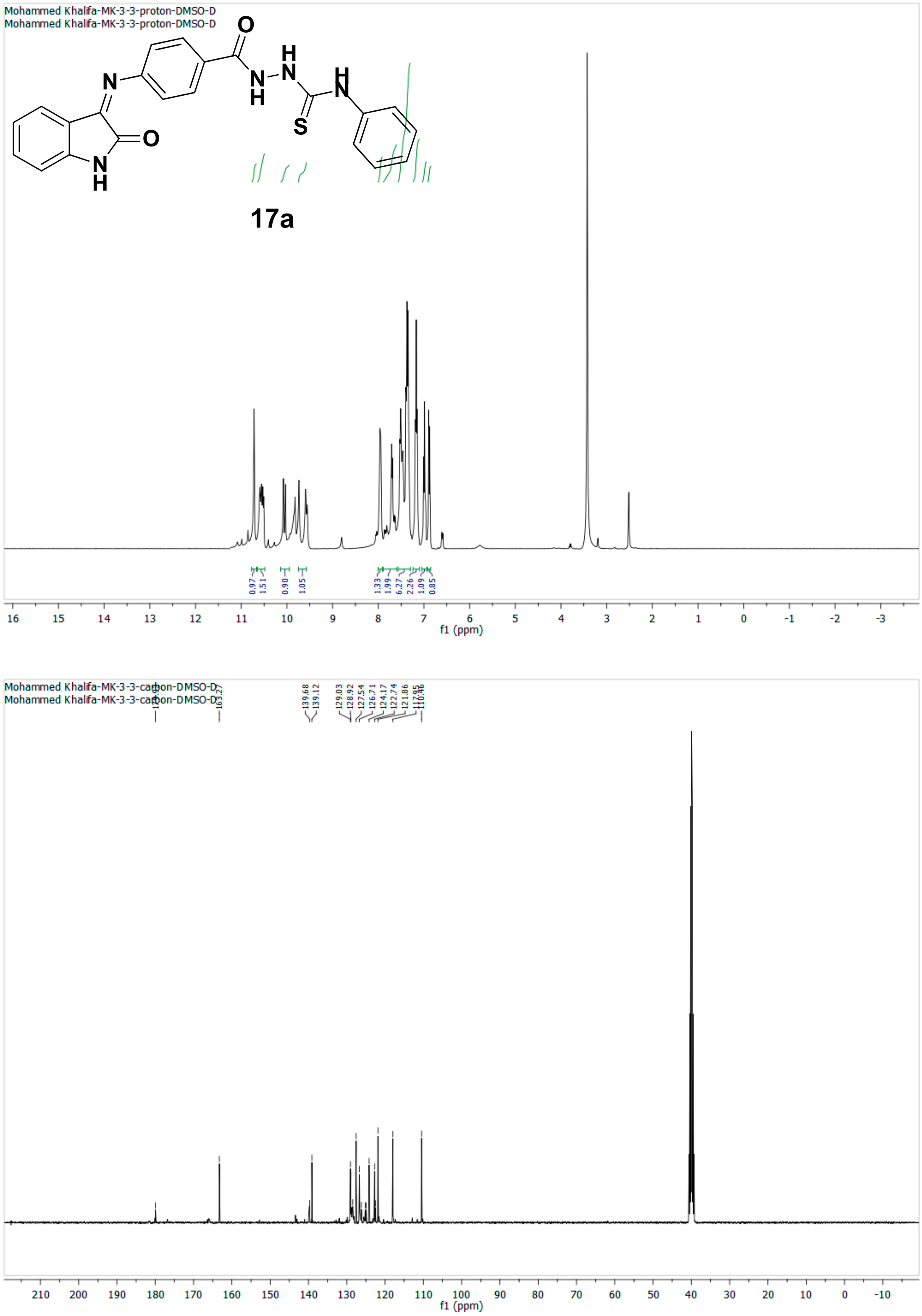

Figure S18. ^{1}H and ^{13}C NMR spectra of compound 17a (DMSO-d6).

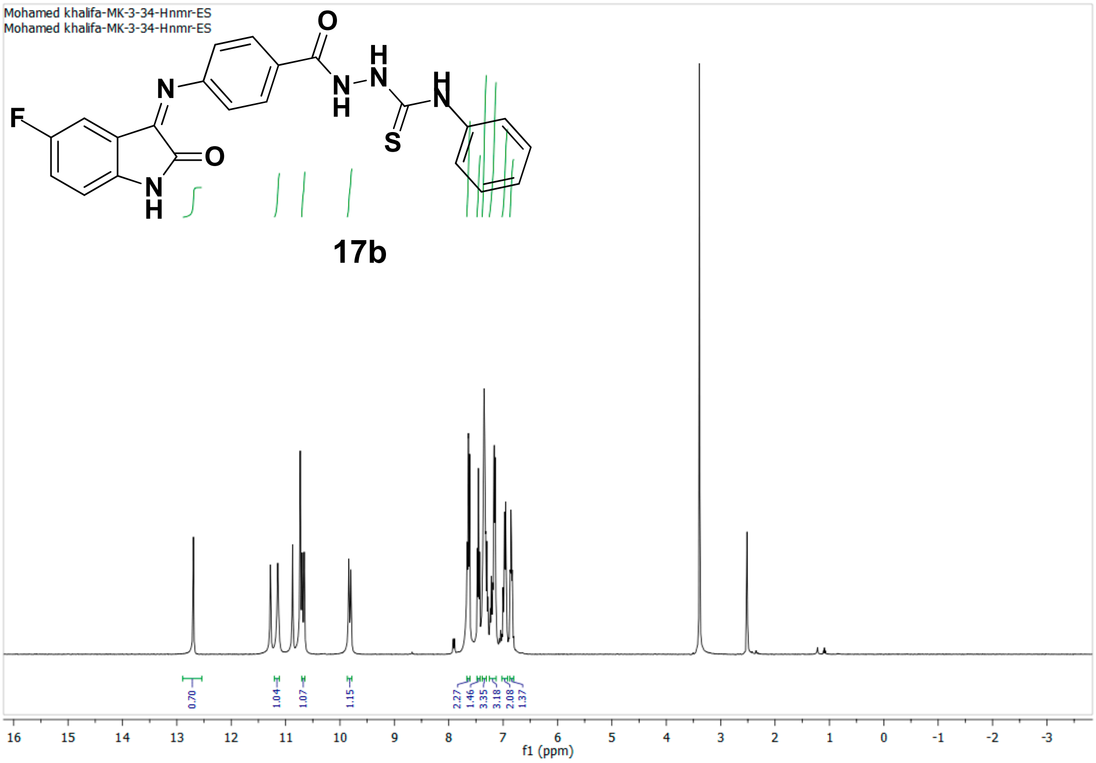

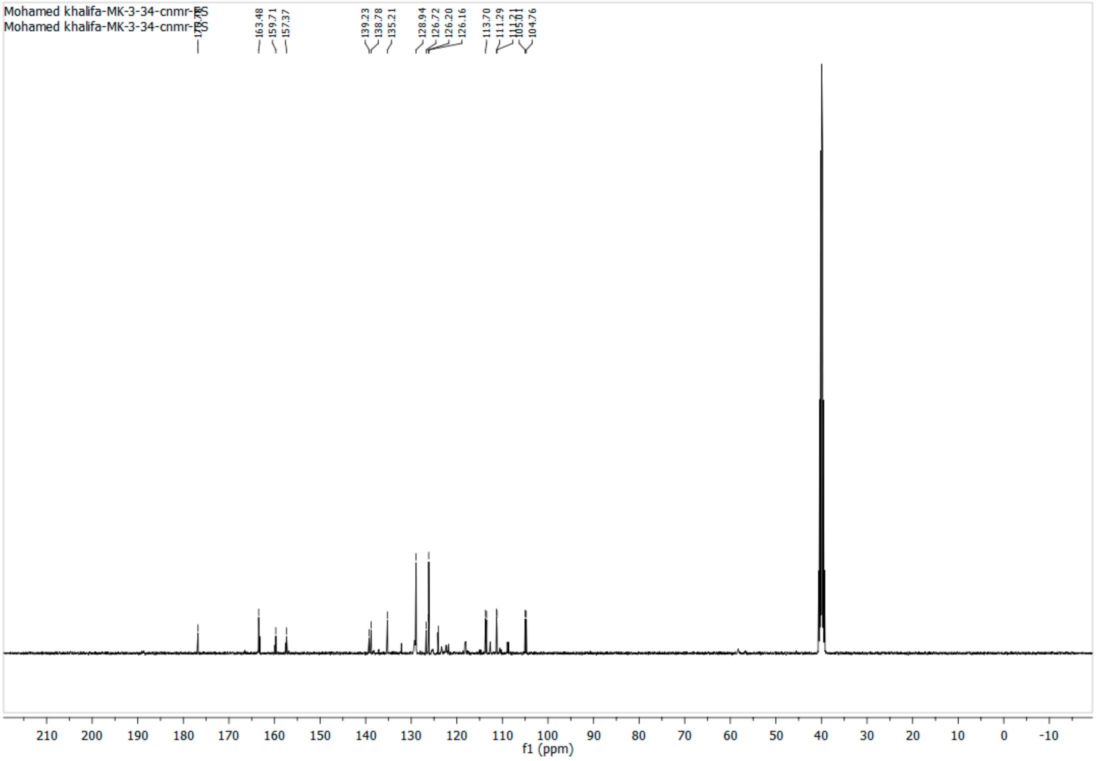

Figure S19. ^{1}H and ^{13}C NMR spectra of compound 17b (DMSO-d6).
